# Changes in the Articular Cartilage Thickness in Patients with Symptomatic Rotator Cuff Tears: A Prospective Study with a Mean 5-Year Follow-Up

**DOI:** 10.3390/jcm13175294

**Published:** 2024-09-06

**Authors:** Jun Kawakami, Nobuyuki Yamamoto, Atsushi Arino, Rei Kimura, Kazuho Aizawa, Hirotaka Sano, Shin Hitachi, Toshimi Aizawa, Eiji Itoi

**Affiliations:** 1 Department of Orthopaedic Surgery, South Medical Center, Ogawara 989-1253, Japan; jun.kawakami.e4@tohoku.ac.jp; 2 Department of Orthopaedic Surgery, Tohoku University School of Medicine, Sendai 980-8574, Japan; atsushi.arino.p3@dc.tohoku.ac.jp (A.A.); rei.kimura.a7@tohoku.ac.jp (R.K.); toshi-7@med.tohoku.ac.jp (T.A.); 3 Department of Orthopaedic Surgery, Obihiro Kosei Hospital, Obihiro 080-0024, Japan; kaz.aizawa1108@gmail.com; 4 Department of Orthopaedic Surgery, Sendai City Hospital, Sendai 982-8502, Japan; sanohirotaka@med.tohoku.ac.jp; 5 Department of Diagnostic Radiology, Tohoku University Hospital, Sendai 980-8574, Japan; hitachi@rad.med.tohoku.ac.jp; 6 Department of Orthopaedic Surgery, Tohoku Rosai Hospital, Sendai 981-8563, Japan; itoi-eiji@med.tohoku.ac.jp

**Keywords:** shoulder, glenoid cartilage change, humeral head cartilage change, rotator cuff tear, osteoarthritis, cuff tear arthropathy

## Abstract

**Objectives:** The purpose of this study was to prospectively investigate the progression of cartilage thinning in patients with symptomatic rotator cuff tears using MRI. **Methods:** Two hundred twenty-five consecutive patients with symptomatic rotator cuff tears visited our institute between 2009 and 2019. Of these, 28 shoulders of 27 patients (mean age, 65 years) who underwent at least two magnetic resonance imaging (MRI) examinations were prospectively enrolled. They all received conservative treatment. The mean follow-up was 67 months. Changes in cartilage thickness and the combined cartilage and subchondral bone thickness at the initial and final MRI were measured using a RadiAnt DICOM-viewer (Medixant, Poznan, Poland). The cartilage thickness of the humeral head was measured in the oblique coronal and sagittal images. The glenoid cartilage was measured in the axial and oblique coronal images. **Results:** At an average period of 5 years, 12 of 28 shoulders (42%) showed more than a 30% decrease in cartilage thickness in the humeral head. The glenoid showed cartilage thinning in only one shoulder (4%). In the humeral head, progressive cartilage thinning was seen mainly in the anterior and posterior parts of the humeral head in the sagittal plane. In the glenoid, progressive cartilage thinning was seen on the entire surface except the posterior area. There was no significant difference in cartilage thickness between the first and final follow-ups for both the humeral head and the glenoid. **Conclusions:** A total of 12 of 28 shoulders (42%) showed more than a 30% decrease in cartilage thickness in the humeral head, which was mainly observed in the anterior and posterior areas of the humeral head.

## 1. Introduction

It has been reported that rotator cuff tears can cause subacromial impingement and inflammation of the capsule, which can lead to an increased rotator cuff tear size and, ultimately, rotator cuff tear arthropathy [1,2,3]. The prevalence of full-thickness rotator cuff tears was reported to be 22% according to the results of the mass screening in one village and 11% in the 20 to 40 age range [4]. Cuff tear arthropathy (CTA) has been reported to occur in 4% of patients with massive rotator cuff tears [5]. In rotator cuff tears, arthritic changes are observed even after a surgical repair, and the incidence of osteoarthritis at a 10-year postoperative follow-up is described as 12% for supraspinatus tendon tears and 50–67% for three-tendon tears except for subscapularis tendons or teres minor tendons [6].

Preventing the progression of osteoarthritis associated with rotator cuff tears includes conservative therapies such as rehabilitation and surgical treatments such as rotator cuff repair. For cases that have progressed to CTA, soft tissue repair, such as superior capsular reconstruction, is considered for mild osteoarthritis, while reverse shoulder arthroplasty is the treatment of choice for those with severe osteoarthritis [7]. The early detection of cartilage changes can prevent cartilage thinning if physicians can perform rehabilitation and rotator cuff repair before it aggravates to severe osteoarthritis. It is minimally invasive for the patient and cost-effective in terms of health care economics.

Magnetic resonance images (MRIs) can detect soft tissue lesions, such as cartilage, ligaments, and synovium, and they are considered useful in the diagnosis of early osteoarthritis [8].

It is still unknown how many of these patients will progress to osteoarthritis. We sometimes encounter cartilage degeneration or defects during arthroscopic surgery even in small or medium cuff tears, which do not show arthropathic changes in X-rays [9]. However, all studies that have investigated the relationship between rotator cuff tears and osteoarthritis of the shoulder used either X-ray or CT, and no studies have evaluated articular cartilage using MRI.

Understanding the progression pattern by capturing early CTA changes that cannot be identified on an X-ray is important in determining the treatment strategy. Therefore, the purpose of this study was to prospectively investigate the progression of cartilage thinning in patients with symptomatic rotator cuff tears using MRI.

## 2. Methods

### 2.1. Subject

Between 2009 and 2019, our institute saw 251 consecutive patients with rotator cuff injuries presenting with symptoms. Of these, 171 patients were prospectively enrolled if they met the subsequent inclusion criteria: (1) a painful rotator cuff tear at the initial appointment, (2) those who underwent at least two MRI exams, (3) those with at least a one-year follow-up, and (4) those who received nonoperative care. The following were the exclusion criteria: (1) those who had a shoulder surgery in the past, (2) those with no shoulder pain at the initial appointment, and (3) those with pain found to be associated with medical conditions other than a rotator cuff tear, such as inflammatory arthritis, glenohumeral arthritis, and cervical spine injuries. Of these, 28 shoulders of 27 patients (12 males and 16 females; mean age, 65 years, range of 48–80 years) who underwent at least 2 MR examinations using the same MR imager were investigated (Table 1). This was because the previous studies showed that changes in cartilage thickness could not be accurately measured unless the same MR imager was used. The two MRI scans were performed at the initial and final follow-up visits. All patients received either nonoperative treatment with medication, subacromial or suprascapular injections, or rehabilitation instructed by a physical therapist. Patients were asked to continue to visit our clinic on a regular basis (at least once every 6 months), even if they were free of symptoms. At the MRI examination, shoulder pain was measured with a 100 mm visual analog scale (VAS), which ranged from 0 mm (no pain) to 100 mm (worst possible pain). Shoulder pain was investigated at rest, during exercise, and at night, and the highest value was recorded as the patient’s shoulder pain. Occupation and comorbidities were also investigated. The present study was approved by the institutional review board of our university.

### 2.2. MRI Examinations

A 3.0 T MRI machine (Intera Achieva 3.0 T, Philips Medical Systems, Eindhoven, The Netherlands) or a 1.5 T high-resolution imaging unit with a microscope coil was used for shoulder MRIs in all patients. For 1.5 T high-resolution imaging, T2-weighted turbo spin echo images (repetition time: 4123, echo time: 90, 3.5 mm slice thickness, 0.35 mm interslice gap, 140 mm field of view [FOV], and 352 × 245 matrix) were obtained. Proton density-weighted turbo spin echo images with spectral presaturation with inversion recovery, repetition time of 179, echo time of 29, 4.0 slice thickness of 0.35 mm interslice gap, 140 mm field of view [FOV], and 320 × 193 matrix were obtained. For 3.0 T images, T2-weighted turbo spin echo images (repetition time, 3500; echo time, 90; 3.5 mm slice thickness; 0.35 mm interslice gap; 140 mm FOV; and 448 × 324 matrix) were obtained. Proton density-weighted turbo spin echo images with spectral attenuated inversion recovery, repetition time of 2800, echo time of 25, 4.0 slice thickness, 0.35 mm interslice gap, 140 mm field of view [FOV], and 512 × 372 matrix were obtained. The registration was performed so that no difference would occur in the measurements between 1.5 T and 3.0 T MRI. These images had a resolution of 0.3 to 0.5 mm.

The position of the shoulder joint was 0 degrees of abduction and 0 degrees of external rotation. The coronal plane was parallel to the direction of the rotator cuff fibres and the humeral shaft axis. The sagittal plane was a plane perpendicular to the coronal plane and parallel to the bony axis. The axial plane was a plane perpendicular to the coronal plane and the humeral shaft axis.

### 2.3. Measurements

**Figure 1:**
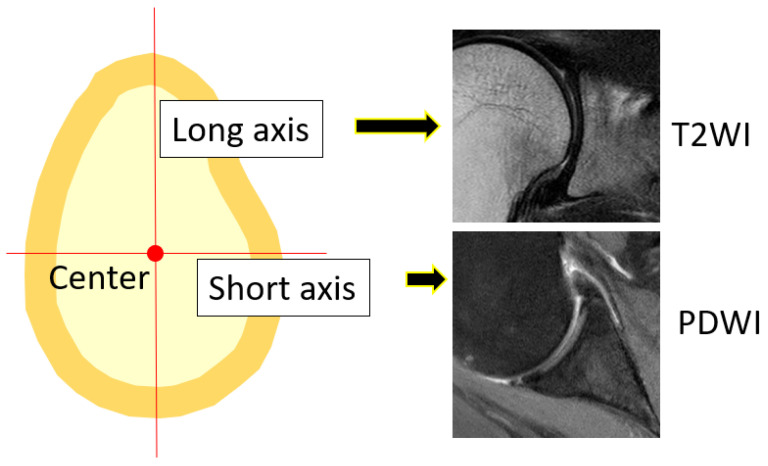
Measurement site and imaging condition of glenoid. T2WI: T2-weighted image, PDWI: proton density-weighted image.

**Figure 2:**
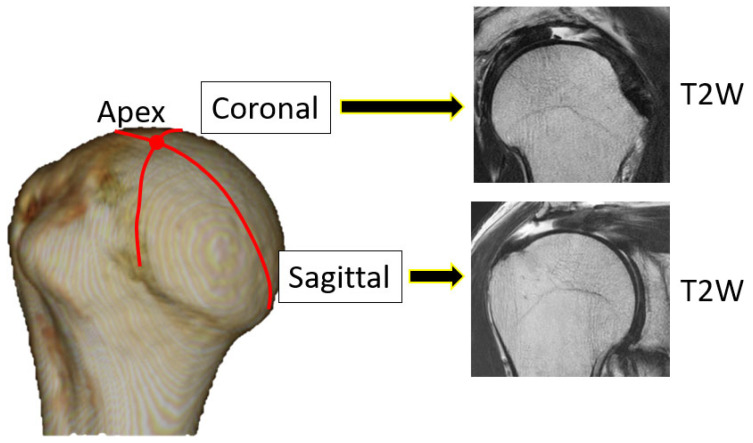
Measurement site and imaging condition of humeral head. T2WI: T2-weighted image.

**Figure 3:**
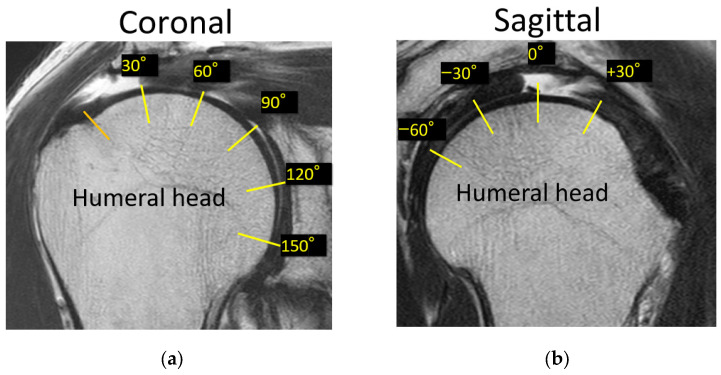
The measurement points of the humeral head. (**a**) In the oblique coronal image, the humeral head was approximated to a circle, and the thickness was measured up to 150 degrees at every 30 degrees from the rotator cuff attachment. (**b**) In the oblique sagittal image, the humeral head was approximated to a circle, and the thickness was measured at 30 degrees anterior, 30 degrees posterior, and 60 degrees from the top of the head. Coronal: oblique coronal image, sagittal: oblique sagittal image.

**Figure 4:**
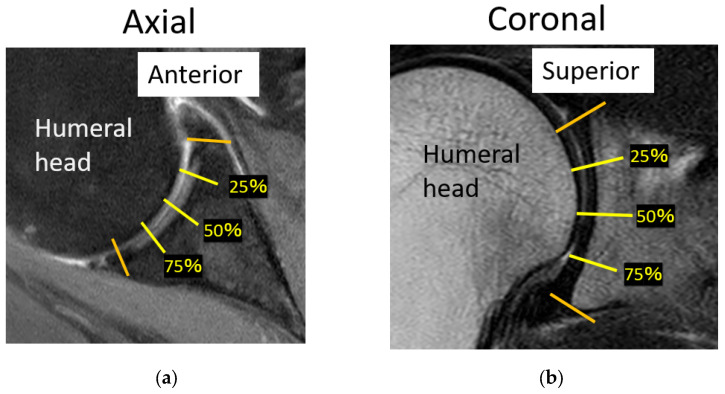
The measurement points of the glenoid. (**a**) The glenoid cartilage was measured from the anterior margin of the glenoid at 25%, 50%, and 75% of the anteroposterior glenoid length in axial view. (**b**) In the oblique coronal image, measurements were taken from the superior margin of the glenoid at 25%, 50%, and 75% of the length from top to bottom.

Tear length, cartilage thickness, and combined cartilage and subchondral bone thickness on the first and final MR images were measured using a RadiAnt DICOM-viewer (Medixant, Poznan, Poland). The rotator cuff tear length was defined from the lateral edge of the rotator cuff attachment to the lateral margin of the rotator cuff. Tear length was categorised based on the classification of DeOrio and Cofield [10]. The thickness of the glenoid cartilage was measured on the long and short axes (Figure 1). A T2-weighted image was used for the long axis, and a proton-weighted image was used for the short axis. The thickness of the humeral head cartilage was measured in the coronal and sagittal planes through the top of the humeral head using T2-weighted images (Figure 2). Since in the T2-weighted images, the cartilage and subchondral bone have a similar signal intensity, the combined thickness of the cartilage and subchondral bone was measured as the thickness. In the proton-weighted fat-suppressed image, the cartilage thickness, which is a high-signal region, was measured. For the humeral head cartilage measurement site, in the oblique coronal image, the humeral head was approximated to a circle, and the thickness was measured up to 150 degrees at every 30 degrees from the rotator cuff attachment (Figure 3). In the oblique sagittal image, the humeral head was approximated to a circle, and the thickness was measured at 30 degrees anterior, 30 degrees posterior, and 60 degrees posterior from the top of the head. The glenoid cartilage was measured from the anterior margin of the glenoid at 25%, 50%, and 75% of the anteroposterior glenoid length in axial view (Figure 4). In the oblique coronal image, measurements were taken from the superior margin of the glenoid at 25%, 50%, and 75% of the length from top to bottom. Since the maximum decrease in the cartilage thickness due to high-demand loaded exercise was 23%, a decrease of 30% or more in thickness was defined as cartilage thinning [11]. The primary outcome was the amount of change in cartilage thickness. The secondary outcome was the areas of cartilage thinning.

### 2.4. Data Analysis

Continuous variables were summarised as mean and standard deviation and compared using Student’s *t* test. *p* < 0.05 was considered significant. All measurements were performed by 2 orthopaedic surgeons (N.Y. and J.K.) who were blinded to each other’s measurements as well as 1 orthopaedic surgeon (J.K.) separated by 4 weeks for inter-rater reliability and intra-rater reliability. The intraclass correlation coefficient (ICC; 2-way mixed effects, single rater, and absolute agreement) was calculated for each outcome for inter- and intra-observer reliability, and 95% confidence intervals (CIs) were reported. For these ICCs, the a priori level of acceptability was 0.75 [12]. All statistical analyses were performed with SPSS Statistics 28 (SPSS Inc., Chicago, IL, USA) and JMP^®^ 15 (SAS Institute Inc., Cary, NC, USA).

## 3. Results

The mean follow-up was 67 months (range of 48–120 months). The average rotator cuff tear lengths were 16 ± 14 mm (2–53 mm) at the first visit and 18 ± 15 mm (4–56 mm) at the final follow-up (Table 2). The data are reported as percentages (n/total) or the mean ± SD (range).

Seven shoulders had a large to massive rotator cuff tear, and 21 shoulders had a small–medium tear. The average VAS value for shoulder pain was 55 mm at the initial visit and 19 mm at the last follow-up. The breakdown of jobs was as follows: 12 people had no job, 7 were light labourers such as desk workers, 6 were medium labourers such as housewives, and 2 were heavy labourers.

The intra- and inter-observer reliability of the measurements were excellent and good, with ICCs of 0.753 (95% CI, 0.536–0.877) and 0.613 (95% CI, 0.210–0.818), respectively. Compared to the first MRI, 12 of 28 shoulders (43%) had more than 30% cartilage thinning of the humeral head at the time of the final MRI, 3 shoulders (43%) had large to massive tears, and 9 shoulders (43%) had small to medium tears (Table 3). Among the 12 shoulders with cartilage thinning, it was observed in the coronal plane in 3 shoulders and in the sagittal plane in 9 shoulders. The glenoid showed cartilage thinning in one shoulder, which was observed both in the coronal and sagittal planes. There was no significant difference in the cartilage thickness at the first visit and final follow-up for the humeral head nor glenoid.

**Figure 5:**
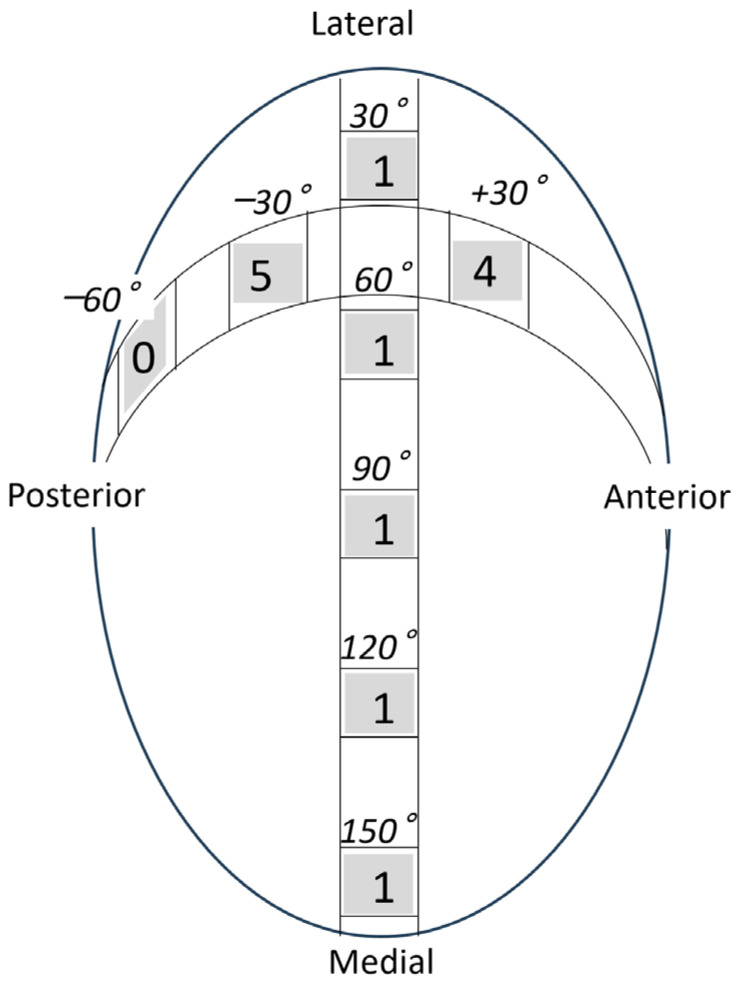
The cartilage thinning area of the humeral head. In the coronal plane of the humeral head, cartilage thinning was observed in 3 of the 28 shoulders, 1 shoulder each at 90, 120, and 150 degrees from the rotator cuff attachment. In the sagittal plane of the humeral head, cartilage thinning was observed in 10 of the 28 shoulders: 4 shoulders at 30 degrees anterior, 5 shoulders at 30 degrees posterior, and 3 shoulders at 60 degrees posterior to the top of the head.

**Figure 6:**
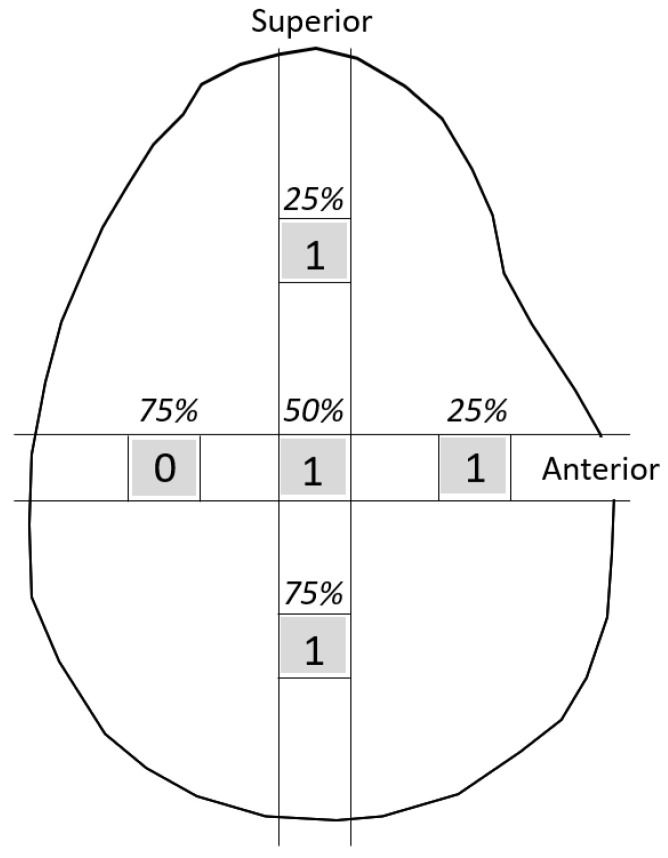
The cartilage thinning area of the glenoid cartilage. Glenoid cartilage thinning was observed in 1 of 28 shoulders. It was observed at 25% and 50% points from the anterior edge in the axial plane and at 25%, 50%, and 75% points from the superior edge in the coronal plane.

In the coronal plane of the humeral head, cartilage thinning was observed in 3 of the 28 shoulders (Table 4; Figure 5 and Figure 6), 1 shoulder each at 90, 120, and 150 degrees from the rotator cuff attachment. In the sagittal plane of the humeral head, cartilage thinning was observed in 10 of the 28 shoulders: 4 shoulders at 30 degrees anterior, 5 shoulders at 30 degrees posterior, and 3 shoulders at 60 degrees posterior to the top of the head. Glenoid cartilage thinning was observed in 1 of 28 shoulders. It was observed at 25% and 50% points from the anterior edge in the axial plane and at 25%, 50%, and 75% points from the superior edge in the coronal plane.

## 4. Discussion

Our data show that at an average follow-up of 5 years, 12 of 28 shoulders (42%) showed more than 30% of cartilage thinning on the humeral head, which was mainly in the anterior and posterior areas of the humeral head and not near the apex of the humeral head. The glenoid showed cartilage thinning in only one shoulder (4%), which was observed over the entire glenoid surface except the posterior area.

Rotator cuff tears cause abnormal glenohumeral joint motion due to a loss of stabilising mechanism provided by the rotator cuff muscles. In addition, rotator cuff tears disrupt the mechanism that maintains negative pressure within the shoulder joint [13]. Because the negative pressure effect within the joint contributes to joint stability, rotator cuff tears have also been reported to decrease shoulder joint stability [14]. This seems to further destabilise the glenohumeral joint. In addition to abnormal joint kinematics, limitation in the shoulder’s range of motion is often seen in patients with a rotator cuff tear. This may decrease the nutritional supply to the articular cartilage. The combined effect of these factors is thought to progress to cuff tear arthropathy [5,9,15,16]. Previous studies have reported that rotator cuff tears affect articular cartilage degeneration and that articular cartilage degeneration is more than twice as common as those without a rotator cuff tear [3,9]. Our results also indicate that 42% of patients with rotator cuff tears showed cartilage thinning during an average of 5 years, which was much higher than we expected.

In our study, progressive cartilage thinning was seen mainly in the anterior and posterior areas of the humeral head, but not near the apex of the humeral head. The reason for this may be that the lower region of the humeral head is used more often during daily living activities and is loaded more than the other areas [3]. The previous study showed that the lower part of the humeral head was in contact with the glenoid fossa when the shoulder was elevated from 0 to 60 degrees. This finding also supports our results [17]. On the glenoid side, progressive cartilage thinning was observed in only one shoulder (4%). Thinning occurred over the entire surface of the glenoid except the posterior area. Cartilage damages in shoulders with rotator cuff tears are reported to be dominant in the posterior humeral head and anterior glenoid [18]. Our results are consistent with a previous study. It was reported that the anteroinferior glenoid and the posterior humeral head were contacted when the upper arm was raised and externally rotated [18]. A rotator cuff tear may aggravate this contact due to a loss of normal kinematics, which may further advance cartilage degeneration.

This study found that cartilage thinning occurred in patients with rotator cuff tears. This proves that it is possible to capture cartilage changes before they progress to severe osteoarthritis. By capturing early cartilage degeneration, rehabilitation and rotator cuff repair might be suggested to prevent cartilage thinning. To make this kind of recommendation, we need to know more about the relationship between cuff repair and cartilage thinning.

## 5. Limitation

There are several limitations in this study. First, the cartilage is a thin tissue (about 2 mm), and given the MRI resolution of 0.3 to 0.5 mm, the precise detection of thinning might be difficult in the present study. The reliability of the measurements in this study, as assessed by the ICC, was 0.75 for intra-observer reliability and 0.61 for inter-observer reliability, suggesting that the errors were within an acceptable range. Second, because imaging is not based on cartilage-specific sequencing, the thickness of the cartilage and subchondral bone is measured, and not the thickness of the cartilage itself. As subchondral bone thickening may occur as the cartilage becomes thinner, the true degree of cartilage thinning may have been greater than we measured. Third, the slices used for the measurements might not be at exactly the same level at the first and final follow-ups, although we did our best to equalise the slice level of each measurement as much as possible. Fourth, the demographic information is limited to age, gender, occupation, and comorbidities without consideration of other relevant factors such as BMI. Fifth, the results are not compared to the progression of cartilage thinning in patients without rotator cuff tears, which limits the ability to evaluate the progression of the condition over time. Finally, the small sample size of 27 patients over five years may limit the generalizability of the findings. Further research with larger sample sizes is needed to validate and extend our findings.

## 6. Conclusions

At an average period of 5 years, 12 of 28 shoulders (42%) showed more than a 30% decrease in cartilage thickness on the humeral head, which was mainly observed in the anterior and posterior areas of the humeral head. On the other hand, the cartilage thickness of the glenoid decreased in one shoulder (4%), which was observed over the entire surface of the glenoid except the posterior area.

## Figures and Tables

**Table 1:** Patient demographics at the first visit.

	Value
Patients	27
Shoulder s	28
Age, mean (range), y	65 (48–80)
Sex, n	
Male	12
Female	16
Follow-up, mean (range), mo	67 (48–120)

**Table 2:** Tear length and size.

	First Visit	Final Follow Up
Tear length ^1^	16 ± 14	18 ± 15
Tear size ^2^		
Small	14	11
Medium	7	10
Large	6	6
Massive	1	1

^1^ Values are presented as mean ± SD in millimetres. ^2^ Tear size categories are based on classification of DeOrio and Cofield.

**Table 3:** Cartilage thickness.

			First Visit	Final Follow-Up	*p*-Value
Humeral head	Coronal ^1^	30°	1.7 ± 0.4	1.7 ± 0.4	0.955
		60°	1.9 ± 0.4	2.1 ± 0.4	0.172
		90°	1.7 ± 0.6	1.9 ± 0.6	0.416
		120°	1.4 ± 0.4	1.5 ± 0.5	0.256
		150°	0.8 ± 0.2	0.9 ± 0.2	0.260
	Sagittal ^2^	+30°	1.2 ± 0.4	1.2 ± 0.3	0.511
		0°	1.8 ± 0.4	1.8 ± 0.5	0.955
		−30°	1.4 ± 0.4	1.5 ± 0.3	0.665
		−60°	0.9 ± 0.3	0.9 ± 0.4	0.885
Glenoid	Axial ^3^	25%	1.9 ± 0.5	2.0 ± 0.5	0.376
		50%	1.3 ± 0.5	1.5 ± 0.5	0.123
		75%	1.6 ± 0.5	1.7 ± 0.4	0.439
	Coronal ^4^	25%	1.9 ± 0.3	1.9 ± 0.4	0.774
		50%	2.1 ± 0.6	2.1 ± 0.6	0.759
		75%	3.0 ± 0.9	3.1 ± 0.9	0.687

The values are presented as the mean ± SD in millimetres. ^1^ The angle from rotator cuff attachment. ^2^ +30°, −30°, and −60° represent the point 30 degrees anterior, 30 degrees posterior, and 60 degrees from the top of the head. ^3^ 25%, 50%, and 75% represent the point from the anterior margin of the glenoid at 25%, 50%, and 75% of the anteroposterior glenoid length in transverse view. ^4^ 25%, 50%, and 75% represent the point from the superior margin of the glenoid at 25%, 50%, and 75% of the Length of top and bottom edges of glenoid in the oblique coronal view.

**Table 4:** Cartilage thinning site and number of shoulders.

	**Glenoid**									
	Axial ^1^				Coronal ^2^					
Measurement site	25%	50%	75%		25%	50%	75%			
Thickness decrease	1	1	0		1	1	1			
	**Humeral head**									
	Coronal ^3^						Sagittal ^4^			
Measurement site	30°	60°	90°	120°	150°		+30°	0°	−30°	−60°
Thickness decrease	0	0	1	1	1		4	1	4	3

^1^ 25%, 50%, and 75% represent the point from the anterior margin of the glenoid at 25%, 50%, and 75% of the anteroposterior glenoid length in axial view. ^2^ 25%, 50%, and 75% represent the point from the superior margin of the glenoid at 25%, 50%, and 75% of the length from the top to bottom. ^3^ The angle from the rotator cuff attachment. ^4^ +30°, −30°, and −60° represent the point 30 degrees anterior, 30 degrees posterior, and 60 degrees from the top of the head.

## Data Availability

Data are contained within the article.
